# The Quality of Online Information for an Uncommon Malignancy—Neuroendocrine Tumours (NETs)

**DOI:** 10.3390/curroncol28010082

**Published:** 2021-02-08

**Authors:** Safa Sohail, Victoria Zuk, Thorvardur Halfdanarson, Dadvid Chan, Sharon Pattison, Ravleen Vasdev, Calvin Law, Julie Hallet

**Affiliations:** 1 Clinical Evaluative Sciences, Sunnybrook Research Institute, Toronto, ON M4N 3M5, Canada; Safa.sohail@outlook.com (S.S.); Victoria.zuk@sri.utoronto.ca (V.Z.); ravleen.vasdev@sri.utoronto.ca (R.V.); calvin.law@sunnybrook.ca (C.L.); 2 Division of Medical Oncology, Mayo Clinic, Rochester, MN 55902, USA; halfdanarson.thorvardur@mayo.edu; 3 Faculty of Medicine, University of Sydney, Sydney, NSW 2006, Australia; dlhchan1@gmail.com; 4 Department of Medicine, Otago Medical School, University of Otago, Dunedin 9016, New Zealand; sharon.pattison@otago.ac.nz; 5 Department of Surgery, University of Toronto, Toronto, ON M4N 3M5, Canada; 6 Susan Leslie Clinic for Neuroendocrine Tumours, Odette Cancer Centre, Sunnybrook Health Sciences Centre, Toronto, ON M4N 3M5, Canada

**Keywords:** neuroendocrine, carcinoid, online information, quality, patient education

## Abstract

Background: Patient information is critical in shared decision-making and patient-centred management for neuroendocrine tumours (NETs). Most adults search the internet for health issues, with over half considering such information to be credible. Therefore, we evaluated the quality of online information on NETs. Methods: Searching for “Neuroendocrine Tumours”, the top 20 websites from Google and top 10 from Yahoo and Bing were identified. Open-access websites written in English were included. Websites indicated as advertisements or directed towards healthcare providers were excluded. Each website was evaluated using the JAMA benchmarks, DISCERN instrument, and the Health on the Internet (HONCode) seal by two independent reviewers. Results: We included 16 unique websites after removing duplicates. Four were education pages from healthcare institutions, 10 were Cancer Society pages, and 2 were general information pages. The average score for JAMA benchmarks was 2.3, with 19% of websites receiving the highest score of 4. Specifically, 31% met the benchmark for authorship, 69% for attribution, 94% for disclosure, and 44% for currency. The average score for the DISCERN instrument was 46.5, with no website achieving the maximum of 80 points. The HONCode seal was present in 3 out of 16 websites (18%). Conclusions: We identified major issues with the quality of online information for NETs using validated instruments. The majority of websites identified through common search engines are low-quality. Patients should be informed of the limited quality of online information on NETs. High-quality online information is needed to ensure that patients can avoid misinformation and actively participate in their care.

## 1. Introduction

Most adults search the internet for health issues, with over half considering online information to be credible [1,2]. While their incidence has been rising and they are now more prevalent than well-known cancers, neuroendocrine tumours (NETs) are not well understood and have multiple potential treatment pathways [3]. Therefore, patient information is critical for shared decision-making and patient-centred management. It is important that NET patients are able to access high-quality websites with recent content; however, the quality of information available to NET patients online is unknown. We systematically evaluated the quality of online information on NETs.

## 2. Methods

We searched for the term “Neuroendocrine Tumours” on Google, Bing, and Yahoo. The top 20 websites from Google and top 10 from Yahoo and Bing were identified. Duplicates were removed. Websites in English that were not password-protected, non-advertisement-based, and not targeted at healthcare providers were included. Quality was assessed using three tools: the JAMA benchmarks, the DISCERN instrument, and the presence of the Health on the Internet (HONCode) seal [4,5,6].

The JAMA benchmarks consist of four domains: authorship, attribution, disclosure, and currency. Authorship refers to whether or not the website has appropriately stated the author’s name, affiliations, and credentials. Attribution identifies whether the website has clearly listed all references, sources, and all relevant copyright information [4]. Disclosure examines whether the website’s ownership, sponsorship, underwriting, commercial funding arrangements, and potential conflicts of interest are present and fully disclosed. Currency refers to displaying the date of publication and subsequent update. Each domain is assigned with either a 1 or 0 depending on whether the website addresses or fails to address that domain, respectively [7].

The DISCERN instrument was designed to help users of consumer health information judge the quality of written information about treatment choices [8]. It evaluates the quality of information about treatment choices using 15 questions addressing the reliability and quality of information. It is scored from 1 (low-quality information with serious shortcomings) to 5 (good-quality information with minimal shortcomings), with a total score of up to 80 points. A 16th question provides an overall quality rating; this question is scored from 1 to 5 and is independent of the total score [5]. This tool can be broken down into three sections. The first addresses the reliability of the publication, indicating whether or not it can be trusted as a source of information about treatment choices. The second set of questions focuses on the quality of information with regards to the treatment choices. The final section is an overall quality rating of the information.

The HONCode seal is displayed on a website if it complies with the standards of the Health on the Internet Foundation’s ethical code for medical websites [6]. The Health on the Internet Foundation was established in 1995 by a panel of experts in telemedicine and medical informatics. It is now the most widely accepted reference for medical websites. The HONCode seal is displayed on the website if the domain complies with the code of conduct and meets the necessary standards.

Website selection and evaluation were done independently by two reviewers (S.S., R.V.), and conflicts were discussed and mediated with a third party (V.Z.). The scoring was reviewed after the first 3 websites’ assessments to ensure consistency.

## 3. Results

Sixteen websites were included after removing duplicates (*n* = 20) and excluding sites aimed at healthcare providers (*n* = 3) and a blog/newsletter (*n* = 1). Included websites are presented in Table 1.

**Figure 1:**
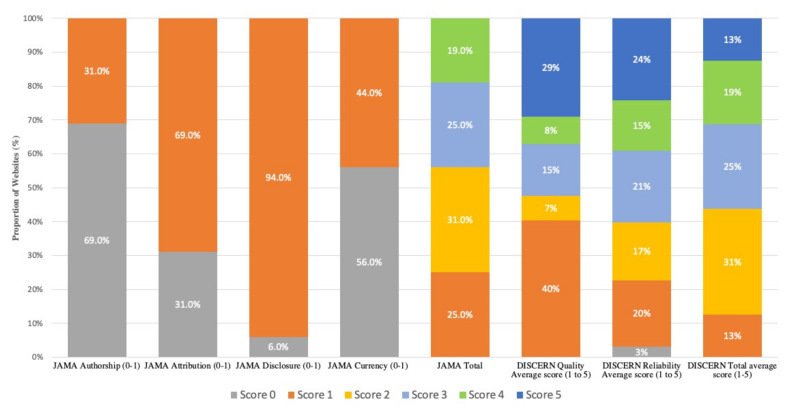
Summary scores for the JAMA benchmarks and the DISCERN instrument (*n* = 16 websites).

The results for the JAMA benchmarks are summarized in Figure 1. The average total score for the JAMA benchmarks was 2.3 (range of observed scores: 1–4). Only 3 out of 16 (19%) websites scored the highest number of 4 points as the total, whereas 4 websites (25%) scored the least number of points of 1. The criteria that most websites did not successfully address was authorship (31%). Disclosure was present and accounted for across most websites (94%).

The results for the DISCERN instruments are detailed in Figure 1. The average score for DISCERN was 46.5 (58%). No website achieved the maximum 80-point score. The highest score achieved was 70 points, and the lowest score achieved was 17 points. For the reliability of information, 24% of websites scored the highest number of points. For the quality of information, 40% of websites scored a 1 out of 5, indicating that these websites lacked any information regarding treatment choices. The question that scored the lowest pertained to what happens if no treatment is selected (question 12), with 14 out of the 16 websites (87%) receiving the minimum score of 1. The second-lowest scored question concerned information on the effect of treatment choices on quality of life (question 13), with 11 out of 16 (68%) websites scoring the minimum score of 1. Regarding the overall quality of the publication as a source of information, 2 out of 16 websites (13%) scored the maximum of 5 points, indicating that they are a useful and appropriate source of information with minimal shortcomings. In contrast, 2 out of 16 websites (13%) scored the minimum 1 out of 5 points, depicting failure to provide good quality information. The most common score for overall quality was 2 points.

The HONCode seal was present in 3 out of 16 websites (19%) and absent in 13 websites (81%). The website that scored the highest on the DISCERN tool with a total score of 70 points did not have the HONCode seal present. The three websites that had the HONCode seal scored 65, 57 and 48, out of 80 points on the summed DISCERN instrument.

Overall, combining the JAMA score and the DISCERN instrument, the top three ranked websites reviewed were #9, 10, and 12 from Table 1. None of these had the HONCode seal. 

## 4. Discussion

Increased accessibility and speed of communication have significantly altered the distribution of information on the internet for health issues [9]. As healthcare providers learn how to enhance shared decision-making and patient engagement, they have to acknowledge that patients are turning towards online sources for information. There is a need to identify the highest-quality information to direct patients towards it. Concerns regarding the low quality of online information for patients have been raised in other cancer sites, such as oesophageal cancer [7]. This is even more important for NETs as an uncommon malignancy with increasing incidence characterized by a chronic course with multiple decision-making points, endocrinopathy impacting quality of life, limited awareness in public and medical communities, and lack of standardized care pathways [10]. Patients with NETs struggle to find disease-specific information; we herein outline that readily available information online is low-quality. Given that health information quality may be circumvented by search engine algorithms, healthcare providers should take an active role in this field, be aware of and refer patients to highest-quality websites about NETs to avoid misinformation, and support informed patient engagement in their care. Higher quality NET-specific websites should be developed in collaboration with patients, care partners, and NET patient advocacy groups. Future work to support this includes qualitative interviews conducted in collaboration with patient partners in order to identify specific needs for shared decision-making, which are currently being conducted.

## 5. Conclusions

We identified major issues with the quality of online information for NETs using validated instruments. While websites providing high-quality information most likely exist, the majority of websites identified through common search engines is of low-quality. Patients should be informed of the limited quality of online information on NETs. High-quality online information is needed to ensure that patients are well informed about their diagnosis and management so they can actively participate in their care.

## Figures and Tables

**Table 1:** Unique websites included in the analysis (in the order in which they appeared in the online search).

GOOGLE
https://www.mayoclinic.org/diseases-conditions/neuroendocrine-tumors/symptoms-causes/syc-20354132 https://www.cancer.net/cancer-types/euroendocrine-tumors/introduction https://rarediseases.info.nih.gov/diseases/13445/neuroendocrine-tumor https://www.cancer.ca/en/cancer-information/cancer-type/neuroendocrine/signs-and-symptoms/?region=on https://www.webmd.com/cancer/neuroendocrine-tumors#1 https://www.cancercenter.com/cancer-types/neuroendocrine-tumors https://www.upmc.com/services/neuroendocrine-cancer/conditions/neuroendocrine-carcinomas https://netrf.org/for-patients/ https://www.cancer.org/cancer/pancreatic-neuroendocrine-tumor/about/what-is-pnet.html https://www.oncolink.org/cancers/carcinoid-neuroendocrine-tumors/all-about-carcinoid-neuroendocrine-tumors https://www.pancan.org/facing-pancreatic-cancer/about-pancreatic-cancer/types-of-pancreatic-cancer/endocrine-pancreatic-neuroendocrine-tumors/ https://www.cancerresearchuk.org/about-cancer/neuroendocrine-tumours-nets http://www.danafarberbostonchildrens.org/conditions/solid-tumors/neuroendocrine-tumors.aspx https://www.cancernetwork.com/gastrointestinal-cancer/neuroendocrine-tumors-of-gastrointestinal-tract https://www.nhs.uk/conditions/neuroendocrine-tumours/
**BING**
https://en.wikipedia.org/wiki/Neuroendocrine_tumor
**YAHOO**
No website returned that was not a duplicate from Google or Bing.
